# Association between Functional Brain Network Metrics and Surgeon Performance and Distraction in the Operating Room

**DOI:** 10.3390/brainsci11040468

**Published:** 2021-04-08

**Authors:** Somayeh B. Shafiei, Zhe Jing, Kristopher Attwood, Umar Iqbal, Sena Arman, Ahmed A. Hussein, Mohammad Durrani, Khurshid Guru

**Affiliations:** 1 Applied Technology Laboratory for Advanced Surgery (ATLAS), Roswell Park Comprehensive Cancer Center, Buffalo, NY 14203, USA; Somayeh.BesharatShafiei@RoswellPark.org (S.B.S.); Zhe.Jing@RoswellPark.org (Z.J.); umar.iqbal@roswellpark.org (U.I.); senaarma@buffalo.edu (S.A.); Ahmed.Aly@RoswellPark.org (A.A.H.); mdurrani20@gmail.com (M.D.); 2 Department of Urology, Roswell Park Comprehensive Cancer Center, Buffalo, NY 14203, USA; 3 Department of Biostatistics and Bioinformatics, Roswell Park Comprehensive Cancer Center, Buffalo, NY 14203, USA; Kristopher.Attwood@RoswellPark.org

**Keywords:** robot-assisted surgery, electroencephalogram, functional brain network, RAS surgical performance

## Abstract

Objective: The aim of this work was to examine (electroencephalogram) EEG features that represent dynamic changes in the functional brain network of a surgical trainee and whether these features can be used to evaluate a robot assisted surgeon’s (RAS) performance and distraction level in the operating room. Materials and Methods: Electroencephalogram (EEG) data were collected from three robotic surgeons in an operating room (OR) via a 128-channel EEG headset with a frequency of 500 samples/second. Signal processing and network neuroscience algorithms were applied to the data to extract EEG features. The SURG-TLX and NASA-TLX metrics were subjectively evaluated by a surgeon and mentor at the end of each task. The scores given to performance and distraction metrics were used in the analyses here. Statistical test data were utilized to select EEG features that have a significant relationship with surgeon performance and distraction while carrying out a RAS surgical task in the OR. Results: RAS surgeon performance and distraction had a relationship with the surgeon’s functional brain network metrics as recorded throughout OR surgery. We also found a significant negative Pearson correlation between performance and the distraction level (−0.37, *p*-value < 0.0001). Conclusions: The method proposed in this study has potential for evaluating RAS surgeon performance and the level of distraction. This has possible applications in improving patient safety, surgical mentorship, and training.

## 1. Introduction

Robot-assisted surgery (RAS) offers advantages such as improved three-dimensionality for surgery, magnified images of the work area, and improved dexterity compared to the traditional surgical framework. While the advantages of RAS are appreciated, the limitations of the robotic user interface and the steep learning curve [1,2] are factors that contribute to a lower utilization of robot-assisted technologies. Even in areas where RAS is widely used, such as gynecology and urology, the outcomes in RAS seem to predominantly correlate with the level of expertise of the individual surgeon [3,4].

Although expertise and performance frequently have been used interchangeably, they do not convey the same meaning in the field of RAS. Even the performance of expert surgeons may be poor in certain surgical situations, i.e., cases with intra-operative challenges [5,6].

A surgeon’s physical and mental performance highly effects patient safety [7]. Several factors in a surgical environment can affect a surgeon’s performance [8,9]. Goodell et al. observed that cognitive distractions lengthen the completion time of laparoscopic surgical tasks [10]. Haptic feedback, a metric that is missed in the current RAS framework, has been frequently mentioned as a key factor influencing surgical performance [11]. It has been proposed that a lack of haptic feedback causes prolonged operative times and learning curves and increases the risk of surgical errors [12,13,14,15], especially those causing tissue damage [16]. In addition, Arora et al. investigated the impact of stressors on performance in the operating room (OR) [17]. They found that the frequent low severity stressors that occurred in the OR were technical-, patient-, and equipment-related problems. In contrast, infrequent yet severe stressors consisted of teamwork-related issues. The occurrence of these situations is associated with an increase in surgeon self-reported stress [17]. Elhage et al. studied individual surgeon performance using three surgical approaches with a simulated task [18], namely, open, laparoscopic, and RAS. In their study, six urological surgeons performed a simulated suturing task utilizing all three types of surgery. They found that in an in vitro model of anastomosis surgery, RAS maintains minimal access and has the accuracy of open surgery with less surgeon discomfort than laparoscopic surgery.

Another factor that contributes to poor surgical outcomes is a lack of training and low skill level. Porter et al. [19] investigated variations in outcomes between patients with rectal cancer treated by expert surgeons trained in colorectal surgery versus non-specialist colorectal surgeons and between surgeons with high- versus low-volume work. The analysis showed that the risk of local recurrence increased, and disease-specific survival was lower in patients treated both by non-specialist colorectal surgeons and by surgeons performing less than 21 procedures during the study. The best result was obtained for the surgeons with specialized training and high surgical practice volumes (10.4% recurrence and 67.3% survival) and worst by surgeons without specialized training and low surgical practice volumes (44.6% recurrence and 39.3% survival) [19].

High performance in multifaceted tasks such as surgery depends upon several factors, including the learner’s ability to develop perceptual, cognitive, and motor skills [20]. The human brain is a complex system that includes various subsystems which interact with each other and dynamically change over different temporal scales while interacting with changes in the environment. It has been shown for motor skill acquisition that there are changes of functional connectivity throughout areas of the brain [21,22].

Several effects on surgical performance and methods for performance evaluation, e.g., subjective and cognitive, have been discussed and proposed in the literature; however, no clinically practical method has been developed and validated for the automatic evaluation of surgical performance. The use of robotics in complicated surgical areas requires close monitoring throughout the operation to ensure that patient safety is maintained [23].

On the other hand, several sources of distraction (defined as “events that cause a break in attention and a concurrent orientation to a secondary task” [24]) in the OR may affect performance. The OR is rich with different sources of distractions, including phone calls, beeper pages, and conversations not pertinent to the surgical procedure [25,26]. Distractions in general can arise from the surgeon’s own personal attitude or from the surrounding OR environment.

In this study, we developed a model that represents the relationship between a surgeon’s performance and distraction level while carrying out RAS surgical tasks in the OR using only surgeon electroencephalogram (EEG) data. There are several metrics that can be derived from EEG data, such as overall spectral features, time–frequency data, and cross-frequency dynamics; however, none of these features express the communication between individual brain areas. We propose the utilization of brain network metrics extracted from EEG data, representing communication and information transformation between brain areas, and extract relationships between those metrics and surgical performance and distraction here.

## 2. Materials and Methods

This study was conducted in accordance with the relevant guidelines and regulations and was approved by Roswell Park Comprehensive Cancer Center Institutional Review Board (I-241913).

**Data Recording Setup**: Utilizing a 128-channel electroencephalogram (EEG) headset (ANT neuro inspiring technology, Inc, Hengelo, The Netherlands) with a frequency of 500 samples/second, EEG data were collected from three robotic surgeons in operating rooms (ORs). EEG data were recorded from 119 brain regions, including the frontal (2 channels), prefrontal (3 channels), central (7 channels), temporal (2 channels), parietal (10 channels), occipital (4 channels), frontal-central (19 channels), frontal-temporal (10 channels), parieto-occipital (17 channels), temporal-parietal (8 channels), and central-parietal regions (18 channels). From the other 9 channels, 2 were reference electrodes placed on the mastoids and 7 electrodes (I1, Iz, I2, CPz, PO5, PO6, and Oz) were excluded from this study due to insufficient signal quality.

**Surgeon’s characteristics and assessment**: All subjects were RAS surgical fellows from the Urology Department at the Roswell Park Comprehensive Cancer Center (Table 1).

A surgery-specific version of the task load index (SURG-TLX) questionnaire was subjectively evaluated by each surgeons and NASA task load index (NASA-TLX) questionnaire metrics were subjectively evaluated by a mentor at the end of each task. SURG-TLX encompasses a multi-dimensional rating of six indices: mental demand, physical demand, temporal demand, task complexity, situational stress, and distraction [27]. The NASA-TLX metrics are mental demand, physical demand, temporal demand, performance, effort, and frustration [28]. Performance scores given by mentor and distraction scored given by surgeon were used in our analyses. The scale of the SURG-TLX and NASA-TLX metrics is 1–20, where 1 is the lowest and 20 is the highest.

**Surgical tasks**: The surgical tasks included in this study were a bladder drop, dissection (lymph nodes, bladder neck, seminal vesicle, peri-ureteric space, lateral pelvic space, anterior rectal space, vascular pedicle, and prostate apex), urethro-vesical anastomosis, and suturing.

**Parcellation of the brain into systems**: Based on the work of [29], regions of the brain and the corresponding recording electrodes located approximately above those regions, were “labeled” as motor-, cognition-, and perception-related brain areas. The “motor process-related areas”, “cognitive process-related areas” and “perceptual process-related areas” were considered in our analyses.

The electrodes located approximately above the motor process-related areas were: “F7”, “F3”, “Fz”,”F4”, “F8”, “FC5”, “FC1”, “FC2”, “FC6”, “C3”, “Cz”, “C4”, “CP5”, “CP1”, “CP2”, “CP6”, “AF7”,”AF3”, “AF4”, “AF8”, “F5”, “F1”, “F2”, “F6”, “FC3”, “FCz”, “FC4”, “FT7”, “FT8”, “TP7”, “TP8”, “FT9”, “FT10”, “AFF1”, “AFz”, “AFF2”, “FFC5h”, “FFC3h”, “FFC4h”, “FFC6h”, “FCC5h”, “FCC3h”, “FCC4h”, “FCC6h”, “CCP5h”, “CCP3h”, “CCP4h”, “CCP6h”, “CPP5h”, “CPP3h”, “CPP4h”, “CPP6h”, “AFp3h”, “AFp4h”, “AFF5h”, “AFF6h”, “FFT7h”, “FFC1h”, “FFC2h”, “FFT8h”, “FTT9h”, “FTT7h”, “FCC1h”, “FCC2h”, “FTT8h”, “FTT10h”, “CCP1h”, “CCP2h”, “CPP1h”, and “CPP2h”.

The electrodes located approximately above the cognitive process-related areas were: “Fp1”, “Fpz”, “Fp2”, “P7”, “P3”, “Pz”, “P4”, “P8”, “POz”, “C5”, “C1”, “C2”, “C6”, “CP4”, “P5”, “P1”, “P2”, “P6”, “PO3”, “PO4”, “PO7”, “PO8”, “PO9”, “PO10”, “P9”, “P10”, “PPO1”, “PPO2 “, “TPP8h”, “PPO9h”, “PPO5h”, “PPO6h”, “PPO10h”, “POO9h”, “POO3h”, “POO4h”, and “POO10h”.

The electrodes located approximately above the perceptual process-related areas were: “T7”, “T8”, “O1”, “O2”, “TPP9h”, “TPP10h”, “TTP7h”, “TTP8h”, “TPP7h”, “TPP8h”, “OI1h”, and “OI2h”.

**EEG signal artifact decontamination**: We used the advanced source analysis (ASA) framework developed by ANT Neuro Inspiring Technology Inc., Netherlands, to pre-process the EEG data. The ASA framework incorporates artifact correction by spatial filtering. It separates brain signal from artifacts based on their topography and subsequently removes artifacts without distorting the brain signal. The separation is determined based on data intervals with a clear artifactual activity as selected by the user and will be used to specify the artifact topography. The method determines which parts of the data are considered brain signals using two criteria: The first criterion specifies the highest permitted amplitude of the brain signal while the second criterion specifies the highest correlation between brain signal and artifact topography permitted. Then, a spatial principal component analysis (PCA) method is used to determine the topographies of the artifact-free brain signals and artifact signals. Finally, the artifact components are removed. It should be mentioned that in the EEG recording system an active shielding technique protects the referential EEG inputs from environmental noise (e.g., grid interference noise and cable movement). Also, by using the “EEGO” software framework for EEG recording, a running DC offset value was calculated per channel over the data. This offset was subtracted from the data to compensate for the DC offset. Line noise artifacts were removed by applying a 60 Hz notch filter to the EEG data. The EEG data from channels were filtered with a band-pass filter (0.2–250 Hz) with a filter steepness of 24 dB/octave. The EEG artifact correction was carried out based on blind source separation and using a topographical PCA-based method. Individual portions of the EEG data were visually inspected for facial and muscular activity artifacts and other artifacts [30]. Then, a spatial Laplacian (SP) technique was applied to the signals and the result was used for feature extraction [31].

**Brain dynamic features**: The brain is parcellated into different areas, including multiple channels [29]. These channels constitute network nodes. Network connectivity was derived here via coherence analysis. These calculations resulted in a weighted connectivity matrix referred to as the adjacency matrix, where the matrix entries represent the connection weights between different areas of the brain (EEG channels). The adjacency matrix was used to extract features of average strength and average search information of “motor process-related areas”, “cognitive process-related areas” and “perceptual process-related areas”.

Strength refers to the total communication weights of channels within each area of the brain. The average strength for each area through each recording was considered for analysis.

Search Information refers to the amount of information (measured in bits) that is required to follow the shortest path between a given pair of nodes [32]. This feature was calculated for pairs of channels within each area and the average value was considered per area in each recording.

We partitioned each adjacency matrix, calculated for every second of each recording, into communities (functional states) using the multilayer modularity maximization criteria [33,34]. The expression of a community refers to the phenomenon where brain regions assigned to the same community are more likely to be strongly connected to one another as compared to regions assigned to different communities [35].

A generalized Louvain-like “greedy” algorithm and Newman–Girvan (NG) null network were used for modularity maximization and community detection [33,34]. A consensus iterative algorithm with 100 repetitions was used to decrease the nondeterministic effect of the community detection algorithm [34,36].

**Figure 1:**
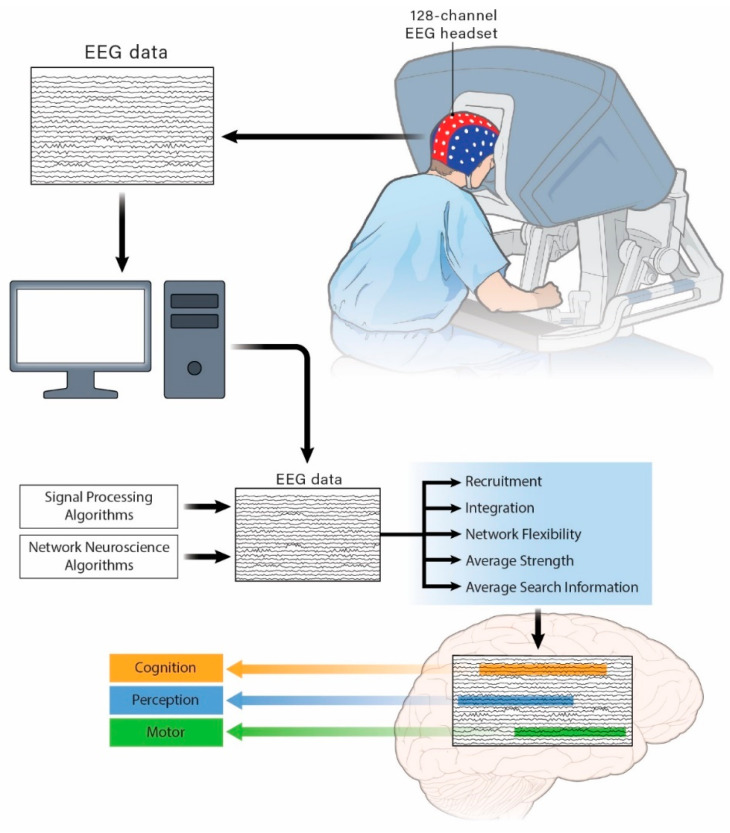
Schematic of the experimental setup and extraction of electroencephalogram (EEG) features within “motor process-related areas”, “cognitive process-related areas”, and “perceptual process-related areas”.

The functional community data assigned each second for each channel were used to extract a module allegiance matrix (MAM) for each recording. The values of the matrix’s elements indicate the probability that two channels can be assigned to the same community in a set of functional brain networks constructed from the recordings. The MAM was used to extract the dynamic brain features of average flexibility, integration, and the recruitment of “motor process-related areas”, “cognitive process-related areas” and “perceptual process-related areas” (Figure 1).

Network flexibility refers to the fraction of times that a channel within an area changes its assigned community in successive one second windows throughout a recording [37]. The average of flexibility for all channels within an area is considered as a feature for each area and recording.

Integration refers to the average probability that a channel is in the same network community as channels from other brain areas. The average integration for every channel within each area is considered a feature assigned to each area and recording [33].

Recruitment refers to the average probability that a channel is in the same network community as other channels from its own area. The average recruitment for all channels within each area is considered a feature assigned to each area and recording [33].

**Statistics:** In our study, we collected EEG recordings from 142 surgeries. The surgeries included more than ten types of urological surgeries. The surgeries were performed by three different surgeons. Surgeon’s EEG was recorded, and surgeon’s performance and distraction were subjectively scored. A total of 14 EEG features were calculated for each surgery. All the EEG features are continuous and described here with the median and inter-quartile range (Q1, Q3). A random intercept model was used to test the differences in performance scores among the different surgeons. We used a general linear model to analyze the relationship between surgeon EEG features and performance score and distraction level.

All tests were two-sided and the statistical significance level was 0.05. All statistical analyses were performed with SAS^®^ (version 9.4, SAS Institute Inc., Cary, NC, USA).

## 3. Results

### 3.1. Performance Evaluation

The median performance score of the 142 surgeries was 12 points with an inter-quantile range of 8 (Q1) to 15 (Q3). The maximum score received (best performance) was 19 points and the minimum score (worst performance) was 1 point. The average score for the 142 surgeries (with the standard deviation) was 11.1 ± 4.2 points (data available in Supplementary Table S1). We did not find any difference between the various surgical tasks (*p* = 0.58). We used a random intercept model to test the differences between surgeons and our results show no differences between surgeons (*p* = 0.38). The univariate analysis results are represented in Table 2.

The EEG features were selected from univariate analysis at a cut-off of *p* = 0.25. Forward model selection was used to detect the effects of the EEG features on the surgeon performance score. In our multivariate analysis, we found that the flexibility of perceptual process-related areas, and strength and recruitment of the cognitive process-related areas were significantly associated with the performance score, where a 0.1 unit increase in the flexibility of perceptual process-related areas lead to an approximate increase of 2.2 for the performance score. Every 10 unit increase in strength for the cognitive process-related areas resulted in an increase of performance of nearly 1 point. A 0.1 unit increase for recruitment of cognitive process-related areas led to a 5-point increase in performance. The results for our final model are represented in Table 3 below.

Our results show that RAS surgeon performance has a relationship with the dynamic features retrieved from the functional brain network.

### 3.2. Distraction Evaluation

Distraction during surgery may be one important factor that can affect a surgeon’s performance score (Pearson correlation coefficient = −0.37, *p* < 0.0001).

We analyzed distraction and its association with EEG features, where distraction was a SURG-TLX metric that was subjectively evaluated at the end of each surgical task with a range of 0 to 20 points, where zero represents absolutely no distraction and twenty is defined as the maximum distraction. For our 142 surgeries, we had 3 missing values and recorded a total of 139 distraction scores. Our median (Q1, Q3) distraction score was 14 (11, 16), while our average distraction score was 13.3 with a standard deviation of 3.1. The minimum distraction was 5 and the maximum was 19.

There were no significant differences between surgeons (*p*-value = 0.17). Because distractions can happen in any surgery, the type of surgery was not considered as a factor in our model.

We continued to use a general linear model to detect the relationship between EEG features and distraction. The results of the univariate analysis between EEG features and distraction scores are shown in Table 4.

Our multivariate results show that every 0.1-unit increase in the integration of perceptual process-related areas was associated with a 1.9-point increase in distraction. Every 0.1-unit increase of the recruitment of perceptual process-related areas was associated with a 1.2-point lower value for distraction. The other EEG features were not found to be significantly related to distraction (Table 5):

## 4. Discussion

The development of an objective model for the evaluation of a RAS surgeon’s performance and distraction level can ensure that a surgeon is operating with high performance and is cognitively engaged, thus improving patient safety. In this study, we have extracted dynamic features from a functional brain network that was developed utilizing EEG data for three RAS fellows performing surgery in the OR.

**Performance evaluation**: Our model shows that both the flexibility of perceptual process-related areas and the strength and recruitment of cognitive process-related areas have a significant relationship with surgeon performance in the OR. In contrast, none of the features extracted from the motor process-related areas had a significant relationship with performance. We found that the network flexibility for perceptual process-related areas has a significant relationship with surgical performance. These promising results suggest the possibility of using these metrics to evaluate RAS surgeon performance in the OR.

**Distraction evaluation**: The working memory capacity of an individual is limited in terms of processing information (cognitive load) [38,39]. Continuous practice during learning maximizes the recruitment of working memory, which frees a portion of the working memory for processing new information, thus enhancing the ability to acquire new skills [38]. Cognitive load theory assumes that optimal learning occurs when trainees have enough cognitive resources devoted for learning (germane), comprehending instructions and noise filtering (exogenous), and finally those available to perform the task (intrinsic) [40]. During learning, exogenous load becomes limited and germane load is maximized, along with the management of the intrinsic load [41]. Therefore, in complicated tasks such as RAS surgery, the addition of multiple factors (including distractions) is associated with increased cognitive load which subsequently influences learning [42,43].

- The development of surgical skill progresses through the following three stages [41,44,45]:
- The “cognitive” stage: Trainees initially learn a skill and thoughtfully perform it.
- The “the associative” stage: With practice, trainees become less thoughtful about the steps required for a skill and can operate with fewer disruptions.
- The “autonomous” stage: The trainee can perform automatically without much thought, meanwhile paying more attention towards other aspects of surgery.

Distraction management is also a skill that should be acquired throughout the RAS surgical skill acquisition process, and dealing with distractions alongside performing surgical tasks is complicated, especially for surgeons-in-training who have not yet automatized their surgical skills (including distraction management) [46]. Our findings also show that distraction is associated with a higher integration and lower recruitment of perceptual process-related areas, confirming that in comparison to surgeons, surgical trainees demand greater perceptual capacity to handle a given surgical task. Hence, distraction negatively affects fellow performance and our results also confirm this (−0.37, *p* < 0.0001).

**Proposed method and implications**: The proposed method has potential to support performance and distraction evaluation in surgery; however, since the proposed method with the existing framework is not processed in real-time, it is required that the application validity of results produced using fewer EEG data is explored and an advanced version of this method is developed for real-time applications.

**Strength of the study**: This study has introduced dynamic features retrieved from the functional brain network that have significant relationships with RAS surgeon performance and distraction while executing risky and complicated surgical tasks in the OR. Upon validation in more general surgical areas and with a wider array of subjects and tasks, the proposed method will improve surgical performance evaluation and will result in improved patient safety in future OR surgeries.

**Limitations of the study**: The ground truth performance and distraction scores were assessed subjectively at the end of each task performance. This increased the subjective effect of the ground truth evaluation and scores used in model development. In addition, the number of participating RAS fellows was limited to only three here. Collecting data from more fellows in various surgical disciplines is necessary to be able to investigate the validity of the developed model in broader applications.

**Future studies and recommendations**: We have used the term “functional brain network” for relating to “motor process-related areas”, “cognitive process-related areas” and “perceptual process-related areas” since these areas could be related to putative functional brain networks; however, the exact groupings that we used may not firmly correspond to such networks. This is a matter that requires further investigation and should be left open to future work.

Another future goal is to gather EEG data from additional RAS fellows while performing diverse surgical tasks in various surgical specialties to validate the proposed method for broader applications. We will also use more data to investigate how distraction affects performance.

## 5. Conclusions

In this study, we have found that the dynamic features extracted from the functional brain networks of surgeons using RAS can be used to evaluate surgical performance and distraction.

## Figures and Tables

**Table 1:** Demographics. RAS: Robot-assisted surgery; OR: operating room.

Subjects Characteristics	Characteristic Options	Number of Subjects (%)
Age (mean)	30–45 (38)	3 (100)
Dominant hand	Right	3 (100)
Gender	Male	3 (100)
Experience	No prior experience in RAS in the OR	3 (100)
Overall years of surgical training/practice	5–10	2 (67)
	10–15	1 (33)

**Table 2:** Univariate analysis results representing the relationship between performance and EEG features.

Variable	Estimate	95% Confidence Interval	*p*-Value
Flexibility motor process-related areas	11.90	(1.28, 22.52)	0.028
Flexibility cognitive process-related areas	15.07	(2.6, 27.53)	0.018
Flexibility perceptual process-related areas	19.50	(7.2, 31.79)	0.0021
Strength motor process-related areas	0.10	(0.04, 0.15)	0.0005
Strength cognitive process-related areas	0.10	(0.05, 0.16)	0.0004
Strength perceptual process-related areas	0.11	(0.05, 0.17)	0.0004
Integration motor process-related areas	26.77	(7.65, 45.89)	0.0064
Integration cognitive process-related areas	28.63	(8.88, 48.38)	0.0048
Integration perceptual process-related areas	39	(13.77, 57.01)	0.0015
Recruitment motor process-related areas	37.12	(13.34, 60.89)	0.0024
Recruitment cognitive process-related areas	48.16	(25.67, 70.65)	<0.0001
Recruitment perceptual process-related areas	8.99	(−1.47, 19.45)	0.0915
Search information motor process-related areas	0.44	(0.16, 0.73)	0.0026
Search information cognitive process-related areas	0.52	(0.22, 0.82)	0.0009
Search information perceptual process-related areas	0.54	(0.19, 0.89)	0.0028

**Table 3:** Result of the multivariate linear analysis, representing EEG features associated with RAS surgeon performance.

Dependent	Parameter	Estimate	*p*-Value
Performance	Intercept	−16.78	0.0011
Performance	Flexibility perceptual process-related areas	21.57	0.0001
Performance	Strength cognitive process-related areas	0.10	<0.0001
Performance	Recruitment cognitive process-related areas	50.36	<0.0001

**Table 4:** Results of univariate analysis, representing the relationship between distraction level and EEG features.

Variable	Estimate	95% Confidence Interval	*p*-Value
Flexibility motor process-related areas	−6.66	(−14.49, 1.17)	0.0948
Flexibility cognitive process-related areas	−10.90	(−20.02, −1.79)	0.0194
Flexibility perceptual process-related areas	−11.15	(−20.27, −2.03)	0.0170
Strength motor process-related areas	0.02	(−0.02, 0.06)	0.3148
Strength cognitive process-related areas	0.02	(−0.02, 0.07)	0.3038
Strength perceptual process-related areas	0.02	(−0.03, 0.07)	0.3622
Integration motor process-related areas	15.64	(1.33, 29.95)	0.0324
Integration cognitive process-related areas	15.79	(1.01, 30.58)	0.0365
Integration perceptual process-related areas	15.39	(−0.93, 31.71)	0.0644
Recruitment motor process-related areas	1.69	(−16.3, 19.67)	0.8531
Recruitment cognitive process-related areas	−5.02	(−22.52, 12.47)	0.5712
Recruitment perceptual process-related areas	−10.30	(−17.89, −2.72)	0.0081
Search information motor process-related areas	0.16	(−0.06, 0.38)	0.1608
Search information cognitive process-related areas	0.17	(−0.07, 0.41)	0.1553
Search information perceptual process-related areas	0.09	(−0.18, 0.36)	0.4958

**Table 5:** Result of multivariate linear analysis, representing EEG features associated with the RAS surgeon distraction level.

Dependent	Parameter	Estimate	*p*-Value
Distraction	Intercept	13.71	<0.0001
Distraction	Integration perceptual process-related areas	19.35	0.0185
Distraction	Recruitment perceptual process-related areas	−11.75	0.0025

## Data Availability

Data is contained within the article or supplementary material. The data presented in this study are available in Table S1.

## Supplementary Materials

The following are available online at https://www.mdpi.com/article/10.3390/brainsci11040468/s1, Table S1: Extracted EEG features from recordings used in this study. Data required for extraction of results. (Excel sheet).
